# Potential role of physical labor and cultural views of menstruation in high incidence of pelvic organ prolapse in Nepalese women: a comparative study across the menstrual cycle

**DOI:** 10.3389/fmed.2024.1265067

**Published:** 2024-02-29

**Authors:** Yvonne Biswokarma, Karen Brandon, Everett Lohman, Ryan Stafford, Noha Daher, Jerold Petrofsky, Uma Thapa, Lee Berk, Robert Hitchcock, Paul W. Hodges

**Affiliations:** ^1^Allied Health Department, Loma Linda University Heath, Loma Linda, CA, United States; ^2^School of Health and Rehabilitation Sciences, Queensland University, St Lucia, QLD, Australia; ^3^Scheer Memorial Adventist Hospital College of Nursing, Banepa, Kavre, Nepal; ^4^Biomedical Engineering Department, University of Utah, Salt Lake City, UT, United States

**Keywords:** chhaupadi, menstratution, prolapse, nulliparous, pelvic floor

## Abstract

**Introduction:**

Pelvic organ prolapse (POP) is a significant health concern for young Nepali women, with potential risk factors including pelvic floor trauma from vaginal delivery and heavy lifting. The prevalence of symptomatic POP (SPOP) among nulliparous women in Nepal is 6%, while the general population of Nepali women aged 15–49 years reports a prevalence of 7%. Surprisingly, the average age of SPOP onset in Nepal is 27 years, challenging the assumption that postmenopausal age and vaginal delivery are the sole risk factors. This study aims to investigate the influence of increased intra-abdominal pressure (IAP) during lifting tasks on pelvic organ descent in Nepali women across different menstrual cycle stages.

**Methods:**

The study included 22 asymptomatic Nepali women aged 18–30 years who regularly engage in heavy lifting. Intra-abdominal pressure was measured intra-vaginally during typical and simulated lifting tasks, which encompassed various scenarios such as ballistic lifting, ramped lifting, and pre-contraction of pelvic floor muscles, as well as coughing, Valsalva maneuver, and pelvic floor contractions. Pelvic floor displacement was recorded using transperineal ultrasound during menstruation, ovulation, and the mid-luteal phase.

**Results:**

Results indicated that pelvic floor displacement was greater during menstruation than ovulation when performing a simulated ballistic lifting task (6.0 ± 1.6 mm vs. 5.1 ± 1.5 mm, *p* = 0.03, *d* = 0.6). However, there was no significant difference in pelvic floor displacement during lifting when the pelvic muscles were pre-contracted.

**Conclusion:**

These findings suggest that lifting heavy loads during menstruation may increase the risk of stretching and injuring pelvic floor supportive tissues, potentially contributing to SPOP in young Nepali women. Pre-contracting pelvic floor muscles during lifting tasks may offer a protective effect. Understanding these factors could aid in developing targeted preventive measures and raising awareness about the impact of heavy lifting on pelvic floor health among Nepali women.

## Introduction

Pelvic organ prolapse (POP) typically occurs when the passive and active pelvic support structures (e.g., tissue, ligaments and muscles) become weakened, stretched, or ruptured generally subsequent to effects of vaginal delivery and menopause. Without support, the pelvic organs can descend under pressure into the vaginal canal and in advanced cases, protrude completely outside the introits (1). Symptomatic pelvic organ prolapse (SPOP) occurs when pelvic organs become so displaced that pelvic function is affected (2). Women report a feeling of something coming out of their vagina, or uncomfortable pressure that can be very painful and even make sitting and other daily tasks difficult (3, 4). SPOP is a substantial problem in Nepal, poorly treated, and affects women at a younger age than in western societies (3, 5–7).

In Nepal, Amnesty International deems SPOP a human rights’ issue because of its serious impact on quality of life (4, 5, 8–10). Although, 10% of Nepali women in the general population have pelvic organ prolapse (POP), the prevalence is 7% during their reproductive years (ages 15–49), and most worrying, 6% of women in the same age category who have never been pregnant report SPOP (11). Furthermore,14% of the women who developed SPOP did so in their teenage years. The mean age of onset for all cases of SPOP in Nepal is 27 years (6). This early onset contrasts the situation for western women who typically develop SPOP after menopause (7, 12). Many women in Nepal who experience the devastating condition of SPOP do not seek help due to embarrassment, inability to pay, or lack of information regarding treatment options (4). The major treatment offered for SPOP to women in Nepal is vaginal hysterectomy, which has major impact for young women of reproductive age. The alternative is pessary use, which has poor patient compliance (13).

In western women, risk factors for SPOP include vaginal deliveries, poor pre- and post-natal care, position at delivery and menopause, leading to weakness of supportive connective tissue and damaged pelvic floor muscles (9, 14–18). These factors cannot fully account for the nearly identical rates of SPOP among nulliparous and parous women of the same age in Nepal. Although genetics and aberrant connective tissue have been implicated, an additional explanation may relate to the influence of variation in estradiol concentration on tissue laxity across menstrual cycle (MC) and the timing of heavy physical activity which challenges pelvic support secondary to high intra-abdominal pressures (7, 19–22). Other biomechanical risk factors have been investigated they were based on interviews not direct measurements during activity and did not control for menstrual cycle phase (23).

### Effects of the menstrual cycle on tissue properties

Low estradiol concentration has been shown to increase musculotendinous stiffness and decrease elasticity of tissues in the lower limb during menstruation (21, 24–27). This has been extensively studied in the peripheral joints such as the anterior cruciate ligament (ACL) of the knee and the plantar fascia of the foot, and in terms of injury rates (21, 28). The functional integrity of the passive and active support structures of the pelvic floor are important to understanding disorders (29). The effect of cyclical variation in hormone concentrations on elasticity/compliance of the pelvic support mechanisms throughout the MC is quite likely as both passive and active support structures of the pelvic organs have receptors for estradiol (and other gonadal hormones) raising the probability of cyclic changes in these tissues (15, 30, 31).

Tissue temperature also affects the mechanical properties of the connective tissues as basal body temperature changes across the MC (0.5–1 degrees lower during early follicular phase than the period after ovulation and the luteal phase) (32, 33). Ligament laxity increases with body temperature when tested across the MC (21). During menstruation, estradiol and temperature are lowest which should lower elasticity and increase stiffness, in connective tissue, with opposite effects during ovulation when estradiol and temperature peak (21).

### Cultural issues related to the MC and physical loading

The physiologic changes that occur throughout the MC may be particularly pertinent in Nepal. A typical workday for a woman living in rural Nepal would include many hours of food preparation, care of children and livestock, as well as religious rituals. Workload for women is 12–22% greater than for men (10). As menstruation is associated with ritual impurity, women are not allowed to cook or perform religious tasks while menstruating, the practice is called “Chhaupadi.” Illegal in Nepal since 2005, it is still widely practiced and it is common knowledge that women undertake a disproportionate amount of their heavier field tasks during monthly menstruation, because they cannot do lighter household work. They are often denied regular food and stay outside the house in makeshift unsafe shelters (8, 34–39) In Nepal, loads are carried in a cone-shaped basket called a “doko” on the back using a head strap called a “namlo” which is worn over the forehead to support the load. This method allows these women to carry very heavy loads long distances through steep and challenging terrain. Women in our study with body weights between 40 and 60 kg typically report that they carry loads between 20 and 80 kg.

### Objectives and hypotheses

The objectives of this study were to: (i) investigate the descent of the pelvic floor during a range of tasks that increase intra-abdominal pressure (IAP), including a simulated lifting task in a group of Nepali women, (ii) compare the pelvic floor descent between tasks performed during three stages of the menstrual cycle, (iii) investigate whether voluntary contraction of the pelvic floor muscles could reduce pelvic floor descent if initiated in advance of a lifting task. We hypothesized that the high incidence of POP in young women may be secondary to excessive heavy lifting during a period when pelvic floor support is suboptimal and that augmented contraction of pelvic floor muscles may enhance support (40, 41).

## Materials and methods

This was a prospective study conducted using a convenience sample of 22 Nepali women. The study protocol was approved by the Institutional Review Board of Loma Linda University Health and by the Nepal Health Research Council. All participants were recruited using a local open advertisement. Prior to enrollment, the researchers, a gynecologist, and orthopedic surgeon screened eligible participants to ensure they met the inclusion criteria. Participants were women aged 18 to 30 years with regular menstrual cycles, who were asymptomatic for POP and engaged in activities that required regular use of a namlo and doko for carrying loads. Exclusion criteria included pregnancy within the last 2 years, pre-menarche, peri- and post-menopausal, SPOP (Stage 2 or greater on Pelvic Organ Prolapse-Quantification System or POP-Q), current irregular periods, use of an intra-uterine device (IUD), and any spinal or orthopedic condition that would affect performance of the lifting tasks (42). Due to the use of Cidex (ASP, Irvine, California) as a disinfecting agent for the Intravaginal Transducer (IVT) sensors, individuals with a history of bladder cancer were also excluded.

Informed consent was read aloud in Nepali to the women because 40% of adult Nepali women have no education (11). A female nurse conducted a intake questionnaire with each participant to ensure that the participant fully understood the study prior to signing the informed consent. The general characteristics of the participants are in Table 1. Each participant attended three separate sessions coinciding with the different phases of the menstrual cycle: within the first 4 days of the onset of menses (early follicular phase), within 24–48 h of ovulation, and 6–10 days later at mid-luteal phase.

**Table 1:** Characteristics of the participants (*N* = 22).

Characteristic	Mean (SD)	No.(%)
Age (year)	27.4 (3.6)	
Body mass index (kg/m^2^)	22.8 (2.8)	
Weight lifted (kg)	19.8 (3.2)	
Number of pregnancies	1.7 (1.0)	
Number of vaginal deliveries*	2 (0,4)	
Number of days since carrying*	2 (1,210)	
Ethnicity		
Newar		5 (22.7)
Chhetri		4 (18.2)
Tamang		5 (22.7)
Brahman		8 (36.4)
Religion		
Hindu		16(72.7)
Buddhist		6(27.3)
Rural		22(100)
Constipation		
No		21(95.5)
Yes		1(4.5)
Menstrual pain		
No		16(72.7)
Yes		6(27.3)
Irregular Periods		
No		16(72.7)
Yes		6(27.3)
Pelvic organ prolapse		
Stage 0		20(90.9)
Stage 1		2(9.1)

*Median (minimum, maximum).

Each participant was provided with a string of Cyclebeads^®^ to assist with tracking their cycle days (CycleBeads^®^ and Standard Days Method^®^ Georgetown University) in addition to instructions and condoms to prevent becoming pregnant during the study. Furthermore, participants were provided with, and educated on the use of, the ClearBlue Advanced Ovulation tests (SPD Swiss Precision Diagnostics GmbH, Petit Lancy, Switzerland) to identify the time of ovulation.

### Procedures

Intra-abdominal pressure was recorded using a validated intra-vaginal pressure transducer (IVT; Department of Bioengineering, University of Utah, Salt Lake City, Utah) and body temperature was recorded by a fast-response sensor (TSD202A, BIOPAC Systems, Inc. Goleta, CA, United States) (43–45). IAP and temperature data were recorded at 1000 Hz using an analog to digital converter (BioPac MP100, Goleta California) with 24 bits of resolution. Data was stored digitally and analyzed later with Biopac Acknowledge 3·9·1 software. The temperature sensor was attached to the IVT with tape at the base of the IVT and covered with a latex-free condom. 2D Ultrasound (US) images for measurement of pelvic floor support/displacement were collected using an ACUSON X300™ ultrasound system (Siemens Healthcare Global, Erlangen, Germany) using a transducer (10,027,930 4C1) placed on the perineum in the mid-sagittal plane (46–48). Placement of the ultrasound transducer was optimized to include the urethral-vesical junction, the anorectal angle and the body of the pubic symphysis as a landmark (46). Elevation with pelvic muscle contraction or descent with increased IAP of the ventral urethral-vesicular junction relative to the mid-point of the pubic symphysis was measured in the position described below.

US data were recorded in video format (frame rate: 30 Hz) using the analog to digital converter system described above.

**Figure 1:**
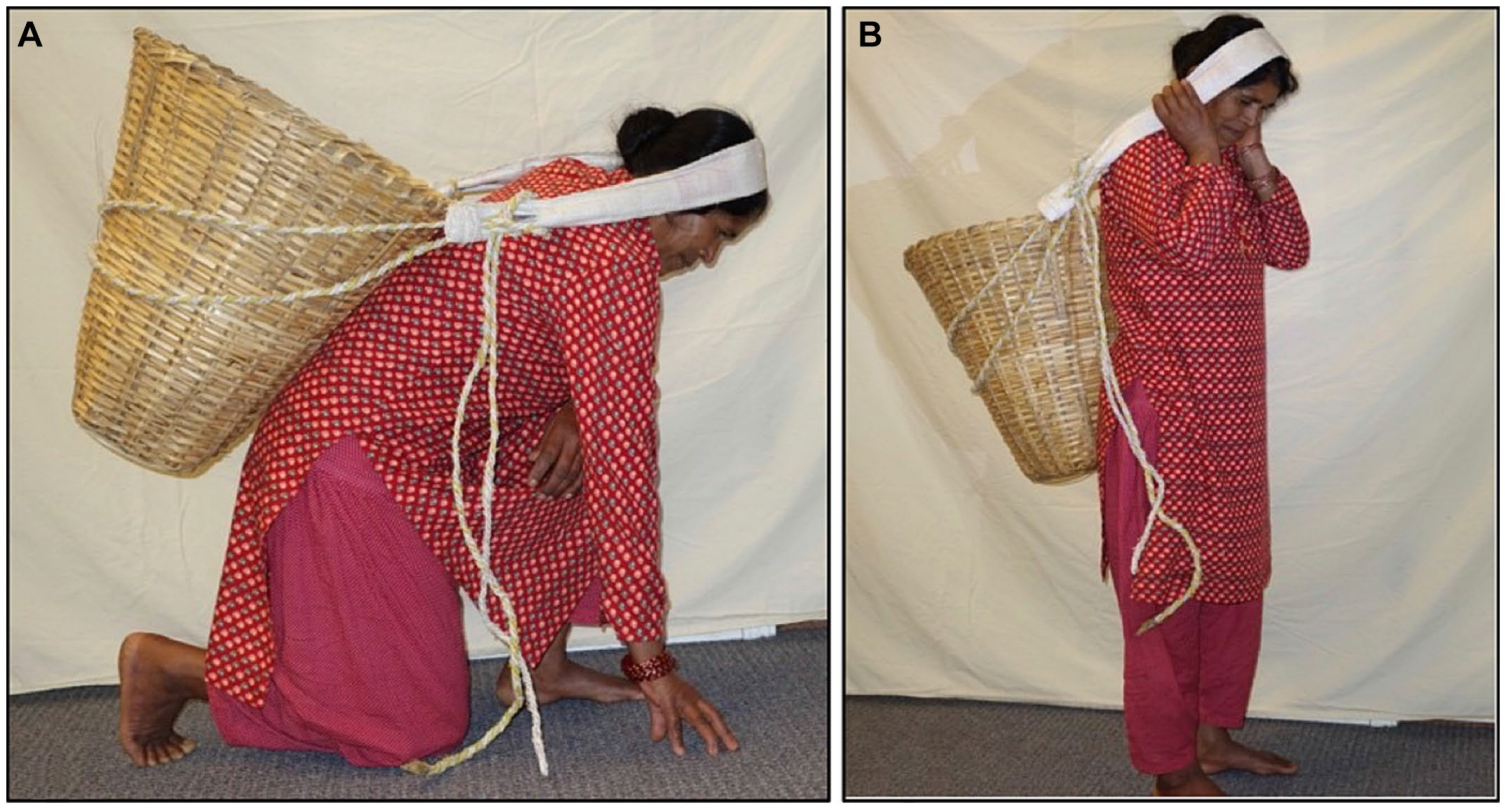
Starting **(A)** and ending **(B)** positions for the doko lift.

Participants were instructed how to insert the IVT into the posterior fornix of the vagina. The positioning was confirmed with US imaging. A baseline resting IAP was recorded with the participants in supine. Prior to commencement of the main trial, measures of IAP generated during a lift of a doko basket were made to identify target pressures for the experimental tasks. Participants performed three trial lifts of a doko basket loaded with a weight that was selected based on the participant’s body weight and subjective tolerance of the load. We aimed to use loads of approximately 40% of the participant’s body weight. If this could not be tolerated by the participant, the load was reduced until successful completion of the lift. The lifted load never exceeded 30 kg, which is the recommended maximum allowable for commercial porters in Nepal (49). The average load lifted by the participants was 19·8 kg. The load was lifted off the ground to a full standing position using a head strap as shown in Figure 1.

Peak IAP was identified from the data recorded for the three repetitions and was averaged to provide a target for the simulated lifting tasks. The peak pressure during the doko lift was only identified in the first session and the same pressure was used as a target for all sessions.

**Figure 2:**
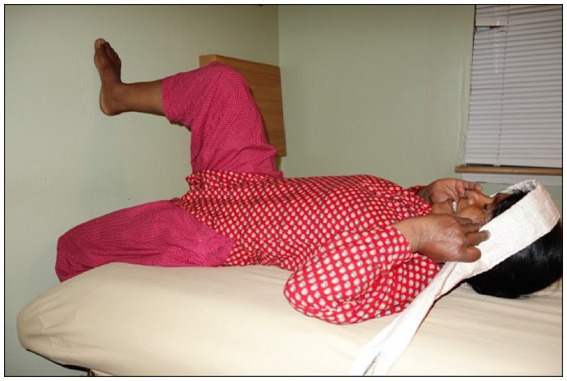
Positioning of participants for ultrasound and pressure data collection for the six tasks.

As it was not possible to make the transperineal US measures of pelvic floor displacement during the doko lift, displacement of the pelvic floor was measured during a series of tasks that increased IAP (three repetitions of each), including a simulated lift task that was designed to emulate the main features of the doko lift. For the simulated lift the participants were positioned on an exam table in supine, approximately 20 cm from a wall. The dominant foot was placed on the wall with 90 degrees of knee flexion; the left leg rested over the end of the table. The head strap was fixed to the bed and placed around the forehead (see Figure 2). The lift was simulated by performing a ballistic isometric head lift against the strap at an effort that approximately matched the IAP generated during the doko lift in standing. Feedback was provided by the examiner to increase or decrease the effort. Participants also performed five other standardized tasks in response to specific verbal instructions (50). These were: (i) a Valsalva maneuver to match the target IAP; (ii) a voluntary cough with moderate effort (not match to target IAP), (iii) a 50% of maximum effort for pelvic floor muscle contraction (not match to target IAP), and (iv) a repeat of the ballistic head lift simulation to matched IAP, but with a 50% of max effort for pre-contraction of the Pelvic floor muscle (PFM). These were chosen to capture functional movements and their effects on the pelvic floor (51).

### Participants

Twenty-seven (27) women completed the informed consent process and were enrolled into the study. Twenty-two were included in the final data analysis. Five were excluded for the following reasons: death in the family preventing attendance for data collection; body mass index over 30 kg/m^2^ with chronic constipation which compromised US measures; failure to complete 2 of 3 sessions; irregular cycles that led to inaccurate timing of ovulation/menstruation; and lack of familiarity with use of the doko lift with a head strap.

Ultrasound images were assessed at rest and at the time of maximum pelvic floor displacement during each task. The ventral urethrovesical junction and the mid-point of the dorsal pole of the pubic symphysis were identified in each image (52). Position of the urethrovesical junction was expressed relative to the pubic symphysis and displacement of the urethrovesical junction was calculated as the length of the vector difference between positions of the junction in the two measures using MATLAB (MathWorks, Natick, Massachusetts, United States). A negative value indicated dorsal-caudal motion (lowering/depression) (53). The peak-to-peak change in IAP was recorded for each trial using AcqKnowledge, software (BIOPAC Systems, Inc. Goleta, CA, United States). Although controlled during data collection, there was some variation in IAP during experimental tasks performed to the target. Only one trial from each task per menstrual phase with recorded pressures that were the closest to the target pressure achieved in an actual dock lift and to each other were included in the final analysis. For the other tasks (cough and PFM contraction), trials were selected that were most similar to each other across the menstrual phases. To aid interpretation of the interaction between IAP and pelvic displacement we calculated an estimate of the tissue compliance as the displacement divided by the change in pressure (mm/cm H_2_O). Although not an accurate measure of compliance, which requires measurement of volume, we considered for the purposes of this study this would aid interpretation of the observations.

### Data analysis

Data analysis was conducted using SPSS Version 28.0. Characteristics of the participants were summarized using means and standard deviations or median (minimum, maximum) for quantitative variables, and frequencies and relative frequencies for categorical variables. The normality of the variables was examined using the Kolmogorov–Smirnov and Shapiro–Wilk tests. A mixed model analysis of variance (ANOVA) was used to determine whether displacement of the pelvic floor, temperature, and IAP differed between the experimental tasks (ballistic lift, voluntary cough, Valsalva maneuver, pelvic floor muscle contraction, and ballistic lift with a pre-contraction of the pelvic floor muscles) and between phases of the menstrual cycle (menstruation, ovulation, and mid-luteal phase). *Post-hoc* comparisons were conducted using a Bonferroni test. One-way repeated measures ANOVA was used to compare mean peak IAP recorded during the doko lift during the experimental tasks. The changes in displacement and IAP between the early follicular and ovulation phases for the same tasks were further compared using a paired t-test, to highlight the difference between the two extremes in the estradiol levels. The level of significance was set at *p* ≤ 0.05.

## Results

Although mean ± standard error of the mean of body temperature recorded during all tasks did not differ significantly between the menstruation, ovulation, and luteal phases (36.8 ± 03 vs. 37.3 ± 0.1 vs. 37.2 ± 0.2, *p* = 0.16), there was a non-significant tendency for a lower temperature during menstruation and higher temperature during ovulation and the luteal phase.

Comparison of pelvic floor displacement between the three menstrual phases failed to reach significance. There was a tendency for differences between the phases of ovulation and menstruation, which represent the extremes in estradiol levels, analyses were repeated without the luteal phase and presented here.

Mean dorso-caudal depression of the pelvic floor ranged between 3.4 mm (ballistic lift with pelvic floor pre-contraction) and 8.5 mm (Valsalva; Table 2). For almost all tasks, the displacement was significantly less during ovulation than menstruation. The exceptions were the Valsalva which involved greater displacement during ovulation (*p* = 0.005), and performance of the ballistic lift with pre-contraction of the pelvic floor muscles which did not differ between menstrual phases (*p* = 0.22). Although displacement was also less in the ovulation phase than menstruation for the PFM contraction, in this case it was elevation rather than depression (*p* = 0.05).

**Table 2:** Mean (SE) displacement (mm) for each task by menstrual phase (*N* = 22).

Task	Ovulation	Menstruation	*p* values
Ballistic*	5.1 (1.5)	6.0 (1.6)	0.03
Ballistic with PFM	3.4 (1.5)	3.9 (2.0)	0.22
Cough*	5.6 (1.5)	6.7 (2.3)	0.02
Valsalva*	8.5 (3.2)	6.0 (2.5)	0.005
PFM*	−3.6 (1.3)	−4.6 (1.6)	0.05

SE, standard error; PFM, Pelvic floor muscle contraction; *p < 0.05 for comparison between ovulation and menstruation.

**Figure 3:**
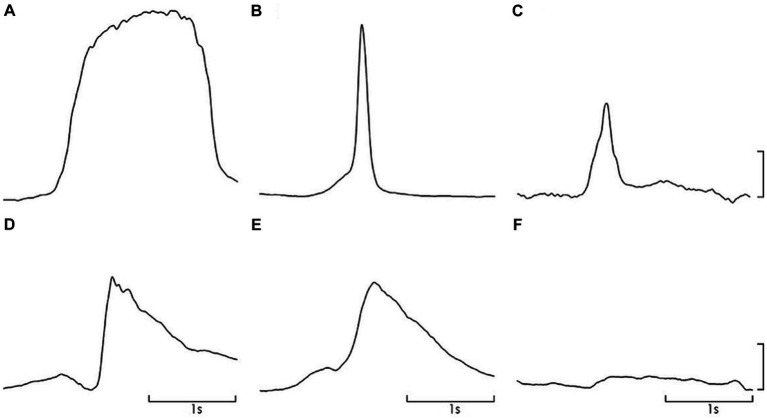
Representative data for IAP change during each task; **(A)**. Valsalva, **(B)**. cough, **(C)**. doko lift, **(D)**. ballistic simulation, **(E)**. ballistic simulation with pre-contraction of pelvic floor muscles, **(F)**. pelvic floor muscle contraction. Scale – 20 cm H_2_O.

Mean ± standard deviation (SD) peak IAP recorded in the natural doko lift was 37·1 ± 4·3 cm H_2_0. As planned in the study, this was not significantly different to that recorded in the ballistic lifts, ballistic lift with a pre-contraction of the PFM, and Valsalva, respectively, (31.6 ± 2.1 vs. 33.3 ± 3.9 vs. 47.3 vs. 4.9); (*p* = 1·00). Peak IAP in the voluntary cough was significantly greater than the natural doko lift (*p* < 0·001) and less during the PFM contraction (p < 0·001). Qualitatively the pattern of IAP increase differed between the experimental tasks. Examples from a representative participant are shown in Figure 3.

Measurements of peak IAP for all tasks and menstrual phases are shown in Table 3. There was no significant difference in the peak IAP change between menstrual phases for the ballistic lifts or ballistic lifts with PFM pre-contraction. Despite analysis of trials being selected as close as possible to that of the target pressure, there was variation between sessions, and there was a consistent and significantly lower peak IAP during menstruation than ovulation for the cough, and PFM contractions (Table 3).

**Table 3:** Mean ± SE of peak intra-abdominal pressure for each task by menstrual phase.

Task	Ovulation	Menstruation	*p* value
Ballistic	30.3 ± 2.0	30.6 ± 2.3	0.86
Ballistic with PFM	29.9 ± 3.3	33.6 ± 4.3	0.27
Cough	73.5 ± 4.0	67.2 ± 3.8	0.02
Valsalva	49.5 ± 4.3	42.2 ± 3.3	0.02
PFM	11.8 ± 2.4	9.0 ± 1.5	0.047

SE, standard error; PFM, Pelvic floor muscle contraction; *p ≤ 0.05 for comparison between ovulation and menstruation.

## Discussion

Descent of the pelvic floor during exertion is greater during menstruation than ovulation, despite similar elevations of IAP. This is relevant for women in Nepal as cultural practice means that women undertake heavy physical labor during menstruation. This challenge to the pelvic floor may pose a risk to the pelvic organ support mechanism and contribute to high incidence of POP among young and even nulliparous Nepali women. We also demonstrated the protective factor of training pre-contraction of the PFM when engaging in activities that increase IAP.

### Pelvic floor displacement/compliance during simulated lifting

Measurement of pelvic floor displacement using transperineal ultrasound imaging during simulated lifting and other functional tasks provided a unique opportunity to measure changes in the biomechanics of the pelvic floor across the menstrual cycle. This method has been used to evaluate support and displacement of the pelvic floor during Valsalva maneuvers, coughing and voluntary contractions (50, 54–56). This is the first application to study control during a functional tasks compared at ovulation and menstruation. Although it would have been ideal to make US measures during a natural lift, this was not possible to maintain image quality during this dynamic activity. Our simulated lift task aimed to replicate the key features of the doko lift (leg position; force application through the head; force applied through feet, matched IAP) and was successful at matching the target IAP which exerts the challenge to pelvic organ support. PFM displacement has been shown to be different in supine versus upright positions, previous work has studied voluntary PFM contractions, but not during exertion (56).

Peak IAP measured in this study was similar to values reported for lifting activities, coughing and Valsalva (45, 52). IAP during the PFM contraction was lower than the other tasks, as the goal is to limit activation of the abdominal muscles and IAP was greater during cough (40). When IAP was low the pelvic floor ascended during the PFM contraction and underwent substantial descent during cough. Notably, our group who had no clinical symptoms of POP were unable to maintain support of the pelvic floor during any task with elevated IAP, and descent of 5.1–6 mm was recorded during the simulated lifting task. Thus, some degree of descent appears characteristic of this task.

Compliance considers the relationship between IAP and pelvic floor displacement. Compliance values were similar to those reported in studies of cough and Valsalva in healthy nulliparous and primiparous woman (54, 55). Compliance varies with task - highest compliance or least stiffness was observed during ballistic lifting (menstruation: 0.2 mm/ cmH_2_O) (Table 4). There are several possible explanations for differences in compliance between tasks. First, net stiffness depends on the sum of the active and passive elements (54). Different compliance would be recorded if the activation of the PFM differed between tasks. This requires consideration. Second, myofascial tissues have viscoelastic properties, which means that stiffness is velocity dependent. Thus, less compliance might be expected when rate of lengthening is greater. Qualitatively, the rate of increase in IAP differed between tasks and is consistent with this proposal compliance was lowest during cough (fastest rate of IAP increase) and highest with Valsalva (slowest rate of IAP increase; Figure 3).

**Table 4:** Pelvic Floor compliance calculations for each task at ovulation and at menstruation in mm/cm H_2_O.

Task	Ovulation	Menstruation
Ballistic	0.17	0.2*
Ballistic with PFM	0.11	0.12
Cough*	0.08**	0.1
Valsalva*	0.17	0.14
PFM*	−0.3	−0.5

*Highest compliance across tasks and menstrual phases, **least compliance, comparable to healthy nulliparous woman 0.083 mm/cm H_2_O (54).

### Pelvic floor displacement/compliance differed between menstrual phases

Our data supports the hypothesis that mechanical properties of the musculotendinous tissues of the pelvic floor would change across the menstrual cycle, secondary to fluctuating estradiol. These findings concur with evidence of changes in mechanical properties of the ACL and plantar fascia with menstrual cycle (21, 22). Unexpectedly, our data infer greater compliance (less stiffness) during menstruation, than ovulation, whereas data for the foot and knee indicate less stiffness at ovulation as opposed to menstruation.

There is not yet consensus regarding the potential role of low estradiol as a risk factor for POP. Dietz argued that increased stiffness of the tissues, with post-menopausal decreases in estradiol, might even be protective against injury from stretch (12). We found the opposite to be true during menstruation—the pelvic floor consistently underwent greater descent during ballistic simulated lifting, despite similar IAP (i.e., greater compliance), during menstruation than during ovulation (7, 12). There are several possible explanations. First, despite greater passive stiffness of the tissue of the PFM during menstruation, the activation of the muscle may be less, resulting in lower net stiffness. The other tissues that have been assessed over the menstrual cycle (ACL and plantar fascia) do not directly include a muscle component and therefore would not be affected by activation changes. This proposal is supported by the contrasting observation in the Valsalva task where greater compliance was recorded during menstruation rather than ovulation. As passive tissue properties would not change between tasks, the likely explanation for different changes in displacement between experimental tasks is variation in muscle activation (57). The IAP profile of the Valsalva task involved a steadier increase, which contrasted the high rate of IAP change in the more ballistic efforts and may have involved a different change in activation. Support of this argument is that pre-activation of the PFM decreased compliance/descent of the PFM (41).

### Implications for pop in women in Nepal

The major implication of this work is that ballistic lifting during menstruation may cause an increased potential for the development of POP secondary to the greater tissue compliance during this phase of menstruation and the biologically plausible argument that repetitive stretching of pelvic support tissues could lead to POP (54). Of potential importance to reduce this risk, pre-contraction of the PFM in advance of the ballistic task reduced compliance/descent by ~30% regardless of menstrual phase. Thus, benefit may be gained to reduce POP risk by using this strategy to reduce ballistic stretch and displacement of the PFM that occurs with increased IAP (58, 59).

Women were able to perform the pelvic floor contractions well with the biofeedback they got from the ultrasound images and verbal feedback provided during our study. Our participants, as those in other studies of this population also seemed eager to learn about self-care regarding preventing SPOP (59, 60). Visual biofeedback methods, like the ultrasound, should be considered in training for pelvic muscle contractions, because written and verbal instruction alone may not be enough (59, 60). All approaches to self-care education need to be culturally informed and appropriate (38).

This study provides a possible starting point to explain the unusually high percentage of POP incidence among young women in Nepal. The next step is to test the mechanism in a longitudinal study and with possible use of elastography and electromyography (29, 61, 62). If shown to be true, PFM strength alone without training in pre-contracting with activities causing increased IAP has not been shown to improve pelvic support, (63).

## Conclusion

Musculotendinous structures are known to be affected by the cyclic changes in estradiol. Although this relationship is complex and not fully understood, the mechanical effects on the pelvic floor can be predicted. This study shows that when estradiol is low during menstruation the pelvic floor is more compliant as opposed to the decreased compliance and higher estradiol present during ovulation. Pelvic floor muscle pre-contraction can compensate for compliance changes across the menstrual cycle with lifting tasks. These findings provide useful information to apply in the prevention of increased repetitive stretching of the PFM a potential risk factor for pelvic floor dysfunction in Nepali women (51).

The authors recommended the following: (i) training in pre-contraction of the pelvic floor muscles when lifting, and while performing other ballistic activities throughout MC and (ii) avoidance of heavy ballistic lifting during menstruation. While developing POP is complex and multifactorial, we believe these recommendations could be useful in prevention of early POP pathology and symptomatology.

## Data availability statement

The raw data supporting the conclusions of this article will be made available by the authors, without undue reservation.

## Ethics statement

The studies involving humans were approved by Institutional Review Board of Loma Linda University Health and by the Nepal Health Research Council. The studies were conducted in accordance with the local legislation and institutional requirements. The participants provided their written informed consent to participate in this study. Written informed consent was obtained from the individual(s) for the publication of any potentially identifiable images or data included in this article.

## Author contributions

YB: Conceptualization, Investigation, Writing – original draft, Writing – review & editing. KB: Conceptualization, Writing – original draft. EL: Conceptualization, Funding acquisition, Methodology, Supervision, Writing – review & editing. RS: Data curation, Formal analysis, Methodology. ND: Formal analysis. UT: Investigation. LB: Methodology. RH: Methodology. PH: Conceptualization, Funding acquisition, Methodology, Data analysis, Supervision, Writing – original draft, Writing – review & editing. JP: Methodology.
